# Fluctuating Disinhibition: Implications for the Understanding and Treatment of Alcohol and Other Substance Use Disorders

**DOI:** 10.3389/fpsyt.2013.00140

**Published:** 2013-10-22

**Authors:** Andrew Jones, Paul Christiansen, Chantal Nederkoorn, Katrijn Houben, Matt Field

**Affiliations:** ^1^Department of Psychological Sciences, University of Liverpool, Liverpool, UK; ^2^Clinical Psychological Science, Maastricht University, Maastricht, Netherlands

**Keywords:** disinhibition, response inhibition, impulsivity, alcohol, motivation, ego depletion, stress

## Abstract

Disinhibition is present in various maladaptive behaviors, including substance use disorders. Most previous research has assumed that disinhibition is a psychological construct that is relatively stable within individuals. However, recent evidence suggests that the ability to inhibit behavior fluctuates in response to environmental and psychological triggers. In this review we discuss some of the factors that cause (dis)inhibition to fluctuate, we examine whether these fluctuations contribute to subjective craving and substance consumption, and we ask if they might increase the risk of relapse in those who are attempting to abstain. The research that we discuss has furthered our understanding of the causal relationships between disinhibition and substance use disorders, and it also highlights opportunities to develop novel treatment interventions. We conclude that substance misusers and their therapists should be made aware of the triggers that can cause disinhibition to fluctuate, and we highlight the need for more research to investigate the effectiveness of inhibitory control training in clinical settings.

## Introduction

The constructs of impulsivity and (poor) executive functioning are central to substance use disorders and other addictive behaviors (1). These constructs are multifaceted and consist of subcomponents which include sensitivity to reward, preference for immediate gratification, risk taking, and disinhibition (2). Disinhibition is defined as the inability to suppress, delay, or change a response that is no longer required or is inappropriate. This inability to control behavior can be measured in the laboratory using computer tasks, such as the Stop Signal (3) and Go/No-Go (4) tasks, both of which require participants to inhibit a dominant motor response. The (in)ability to control oculomotor reflexes is another index of disinhibition: for example, the anti-saccade and delayed ocular return tasks require individuals to suppress or delay reflexive saccades to visual stimuli (5, 6).

Impaired ability to control behavior is specified in at least one of the DSM 5 criteria for substance use disorders [“persistent desire or unsuccessful effort to cut down or control (substance) use”; American Psychiatric Association, 2013]. Consistent with this, there is overwhelming evidence for increased disinhibition (or poor inhibitory control) in substance abusers, alongside elevations in other aspects of impulsivity [for a comprehensive review see (7)]. For example, alcohol-dependent patients perform worse on the Stop Signal task than healthy controls (8–10). Furthermore, different subtypes of alcoholism may show more pronounced disinhibition than others (10, 11). Elevated disinhibition is also seen in abusers of cocaine (12) and methamphetamine (13), and in cigarette smokers (14).

In individuals who drink alcohol but are not dependent, Colder and O’Connor (15) found that an index of disinhibition (commission errors on a Go/No-Go task) was associated with alcohol use. Other studies have also shown that elevated motor disinhibition is associated with problem drinking in community samples of non-dependent drinkers (16–18), although this relationship is not always seen (19, 20). Weafer et al. (6) demonstrated that oculomotor disinhibition predicted heavy drinking in individuals with Attention Deficit Hyperactivity Disorder (ADHD), but not in healthy controls. We direct readers to recent reviews of the relationship between substance use and disinhibition from Verdejo-Garcia et al. (7) and Dick (21).

There is some debate over whether disinhibition is a cause or a consequence of substance use. There are two possible explanations, which are not mutually exclusive: (i) chronic heavy substance use may increase disinhibition through neurotoxic effects in the prefrontal cortex, and (ii) children and adolescents who are particularly disinhibited are at increased risk of initiating substance use and developing substance use disorders. In support of the first explanation, it is well-established that chronic alcoholics present with structural brain damage, with prefrontal areas showing the greatest damage associated with heavy drinking (22, 23). These prefrontal regions are implicated in executive functions, including disinhibition, so structural and functional damage here should correspond to disinhibited behavior (24–26). Furthermore, animal research reveals that chronic alcohol administration and/or binge cycles causes structural and functional damage to the brain (27–29).

On the other hand, individual differences in disinhibition may explain why some adolescents start to use alcohol and drugs at a young age and then develop substance use disorders, whereas others do not. A series of longitudinal studies have demonstrated that increased disinhibition during childhood is a risk-factor for substance abuse in later life (30–32). One study examined the developmental trajectory of behavioral control and demonstrated that children with lower levels and who exhibited slower development (of behavioral control) were more likely to report alcohol-related problems at later time points (33). Other studies have investigated “neurobehavioral disinhibition,” a latent variable derived from questionnaire measures and observer reports of executive cognitive functioning and disruptive behavior and have found that disinhibition at age 12 predicts later substance use behaviors (31, 32). Using a cross lagged model, Fernie et al. (34) demonstrated that disinhibition predicted alcohol use 6 months later in British schoolchildren, although they found no evidence for alcohol-induced impairments in inhibitory control. Furthermore, in adults, disinhibition in heavy drinkers has been shown to predict severity of alcohol dependence at 4 years follow-up (35). Finally, children with ADHD demonstrate disinhibited behavior, and affected children are significantly more likely to develop alcohol dependence (36).

## Does Disinhibition Fluctuate Over Time?

Whilst these prospective studies suggest that elevated disinhibition is a risk-factor for heavy drinking, this does not necessarily imply that disinhibition is purely a trait variable, i.e., one that is stable over time with individuals. On the contrary, there is now solid evidence that disinhibition, like other states, can fluctuate within individuals. In a recent review of the literature, de Wit (2) noted that “… abrupt environmental, physiological, or emotional events may cause transient “state” changes in either self-control or inhibition that may result in re-initiation of drug use” (p.28). In the remainder of this review we will discuss the psychological mechanisms that underlie state fluctuations in disinhibition, and we investigate whether temporary increases in disinhibition are associated with increased subjective craving and self-administration of the substance. We note here that whilst we have attempted to organize this material into distinct categories, there is likely to be considerable overlap between categories and we highlight this at appropriate points in our manuscript.

## The Pharmacological Effects of Alcohol: “Priming”

Acute alcohol intoxication, or alcohol “priming,” leads to increased desire for and self-administration of alcohol (37), effects which are dose-dependent (38). As well as increasing motivation to drink, alcohol priming also leads to changes in cognitive functions, including disinhibition. For example, moderate (0.4 g/kg) to large (0.8 g/kg) doses of alcohol impair inhibitory control on a variety of behavioral measures (39, 40). Furthermore, these deficits are evident at doses too low to disrupt general psychomotor performance, suggesting fairly selective effects of acute alcohol on disinhibition [see detailed reviews from Fillmore (41) and (42)]. More recent research demonstrates that the effects of alcohol intoxication on disinhibition may be particularly pronounced when participants are required to inhibit their responding to alcohol cues (43) [but see (44)], a point that we return to later.

Given that disinhibition is sensitive to acute alcohol consumption, it has been hypothesized that fluctuations in disinhibition may mediate the alcohol priming effect, i.e., the “loss of control” over drinking that occurs following alcohol ingestion (42). This prediction was based on findings reported by Weafer and Fillmore (45), who demonstrated that consumption of 0.65 g/kg of alcohol led to increased commission errors on a Go/No-Go task. Importantly, those authors found that the magnitude of alcohol-induced disinhibition was positively correlated with *ad libitum* alcohol consumption in a subsequent testing session (when participants were sober), with alcohol-induced disinhibition accounting for 20% of the variance in alcohol consumption.

While informative, this study does not tell us if alcohol-induced increases in disinhibition are related to alcohol-induced increases in the motivation to drink alcohol, i.e., if alcohol-induced disinhibition might mediate the alcohol priming effect. In the first study to directly investigate this issue, we (46) found that alcohol administration led to increased *ad-libitum* alcohol consumption, but it did not affect disinhibition (performance on a Go/No-Go task), and disinhibition was unrelated to *ad libitum* alcohol consumption after alcohol administration. In summary, at present there is no convincing evidence to suggest that state fluctuations in disinhibition mediate the alcohol priming effect, although to date only one study has directly tested this hypothesis.

It is also important to point out that disinhibition can fluctuate in sober people, in response to non-pharmacological manipulations, and these manipulations may influence the motivation to drink alcohol and contribute to loss of control over drinking behavior. We review these environmental and psychological influences on disinhibition in the next sections of the paper.

## Ego Depletion

The Limited Resource theory of self-control (47) posits that individuals have a finite reserve of self-control that they can employ to regulate their behavior. If demands on self-control are excessive and/or maintained over a long period of time then this resource will become depleted and subsequent attempts to regulate behavior will be unsuccessful; the state of having depleted self-control resources has been termed “Ego Depletion.” The analogy of self-control as a muscle is often used: in the same way that repeated exertion of a muscle over a short period of time will weaken that muscle and lead to fatigue, self-control capacity will be diminished after exerting self-control for a prolonged period (47, 48). In relation to substance use, individuals may need to engage self-control in order to overcome urges or cravings to use the substance when they encounter substance-related cues. For example, Muraven and Shmueli (49) found that when social drinkers were exposed to alcohol cues, they showed greater disinhibition than when exposed to neutral cues. A similar pattern of results was reported by Gauggel et al. (50) in detoxified alcohol-dependent patients [although a subsequent study failed to replicate this (51)]. One explanation for these findings is that alcohol abusers have to engage self-control resources in order to resist their urge to drink alcohol when exposed to alcohol cues, and this leads to a depletion of self-control resources, which manifests as increased disinhibition. However, it is important to point out that ego depletion is not the only explanation for increased disinhibition after cue exposure, a point that we address in the next section.

As predicted by limited resource theory, alcohol consumers are more likely to drink to excess if they have been exerting self-control. Muraven et al. (52) demonstrated that a self-control task (thought suppression) led to increased alcohol consumption compared to a comparison task that did not require self-control (performing mental arithmetic). Furthermore, this increase in alcohol consumption was evident despite participants being given a financial incentive to limit their alcohol consumption. Comparable findings were reported by Friese et al. (53). The influence of ego depletion was also examined in naturalistic settings by Muraven et al. (54), who used ecological momentary assessment to assess self-control demands and alcohol consumption. They found that on days when individuals reported higher self-control demands (expending more effort in regulating mood, controlling thoughts, or dealing with stress), they tended to drink more alcohol and were more likely to violate self-imposed limits on alcohol consumption [c.f. Limit Violation Theory: (55, 56)]. Combined, these studies suggest that ego depletion can have a causal influence on drinking behavior, and temporarily increased disinhibition (as a consequence of ego depletion) is a plausible explanation for these effects.

To test this, Christiansen et al. (57) conducted a laboratory study to examine whether the effects of ego depletion on *ad libitum* alcohol consumption were mediated by increases in disinhibition, in a sample of heavy social drinkers. Their results demonstrated that ego depletion caused a marked increase in *ad libitum* alcohol consumption and also a slight increase in disinhibition, replicating the effects reported in the aforementioned studies (49, 50). However, increased *ad libitum* consumption after ego depletion was not mediated by changes in disinhibition. Instead, the effects on *ad libitum* consumption were mediated by the perceived *effort* of suppressing emotions and thoughts during the ego depletion manipulation. These findings are consistent with recent evidence demonstrating that ego depletion may not influence behavior because of an actual deficit of self-control resources, but instead because of the perceived effort and beliefs about ego depletion (i.e., the belief that self-control is a limited resource and one can be “at the end of one’s tether”). For example, Job et al. (58) told participants that self-control was unlimited, and these instructions completely eliminated ego depletion effects. This was later replicated, but only in situations of minor ego depletion (59). Similarly, Alberts et al. (60) demonstrated that priming thoughts of persistence led to stable self-control, i.e., immunity from ego depletion effects. Finally, Clarkson et al. (61) showed that individuals who perceive themselves as less (versus more) depleted are somewhat protected against ego depletion effects. Considered together, these studies suggest that ego depletion effects are genuine phenomena, but they may be mediated by beliefs about self-control rather than a directly observable transient change in disinhibition [see also (48)]. The findings from the study by Christiansen et al. (57) demonstrate that this account may also explain the role played by ego depletion in substance use disorders.

## Exposure to Drug-Related Cues (“Cue Exposure”)

As noted in the previous section, disinhibition increases when substance users are exposed to substance-related cues. It is well-established that exposure to substance-related cues elicits a range of responses include physiological changes (such as increased heart rate and skin conductance), increased subjective craving, and increased drug self-administration (62). Some theorists have suggested that fluctuations in disinhibition may partially mediate the effects of drug cue exposure on craving, drug self-administration, and relapse in those attempting abstinence [e.g., (2)].

As we discussed in the previous section, there is compelling evidence that alcohol-related cues lead to increased disinhibition when presented to alcohol-dependent patients [(49, 50); see also (63)]. The pattern of results obtained from studies with non-dependent drinkers is more mixed: both Weafer and Fillmore (64) and Petit et al. (65) demonstrated that alcohol-related pictures caused transient increases in disinhibited behavior. However Nederkoorn et al. (18) found no increase in disinhibition when social drinkers were exposed to alcohol-related pictures (versus other types of pictures), and Jones et al. (66) replicated the methodology of Gauggel et al. (50) in social drinkers and found no change in disinhibition after holding and sniffing an alcoholic drink (compared to exposure to a control drink). This suggests that alcohol-dependent patients may be more vulnerable to fluctuations in disinhibition after exposure to alcohol cues than non-dependent individuals.

Is disinhibition implicated in cue reactivity? Papachristou et al. (67) demonstrated that individual differences in disinhibition (as assessed with a Stop Signal task) moderated cue-provoked craving: individuals exhibiting greater disinhibition showed a greater increase in craving following cue reactivity. In a subsequent study with alcohol-dependent patients, disinhibition predicted 13% of the variance (68) in peak craving following cue exposure. These studies demonstrate that individual differences in disinhibition can moderate the strength of subjective responses to alcohol cues, but they do not tell us if alcohol-related cues lead to increased disinhibition, and if cue-induced increases in disinhibition mediate other aspects of cue reactivity such as subjective craving and drug self-administration. To our knowledge, the only study to investigate this issue was recently performed by our group. We found that alcohol cues increased subjective alcohol craving and self-administration of alcohol, but had no effect on disinhibition; furthermore, disinhibition after cue exposure was correlated with the strength of craving, but was unrelated to alcohol self-administration (66). Therefore, additional research is required to investigate whether state disinhibition might mediate the effects of alcohol cues on craving and drinking behavior.

## Stress, Arousal, and Negative Emotional States

The handful of studies that investigated the effects of stress on disinhibition have yielded contradictory findings. For example, one study demonstrated that acute stress magnified the influence of alcohol-related cues on disinhibition (44), whereas Constantinou et al. (69) found that acute stress actually *reduced* disinhibition in opiate users and matched non-user controls. These findings may be reconciled if we carefully consider the severity of the stress response: Henderson et al. (70) demonstrated increased disinhibition after both high and low stress levels of stress, whereas moderate levels of stress led to reduced disinhibition. Therefore, we can speculate that the relationship between disinhibition and stress may follow a U-shaped function, although it is difficult to characterize and directly compare the severity of stress induced in the Constantinou et al. (69) and Zack et al. (44) studies.

Both negatively valenced and positively valenced stimuli can increase disinhibition. For example, increased disinhibition is seen in response to stimuli depicting painful situations (71), fear and biological threats (72). Positively valenced stimuli have similar disinhibiting effects (73, 74), that may be stronger than those produced by negative stimuli (75). In an interesting parallel to the literature on stress and disinhibition, it seems that the emotional intensity of the stimuli may be an important factor, because both positive facial expressions and (low intensity) threat images can reduce disinhibition, whilst high intensity threat increase it (76). Given that highly valenced images (both positive and negative) are also highly arousing (77), we can speculate that the relationship between arousal and disinhibition may also be U-shaped, much like the relationship between stress and disinhibition. High and low levels of arousal may interfere with the allocation of attention, creating conflict, and competition between inhibitory and attentional processes (74, 78). However, this is speculative and we highlight the need for further research to clarify the relationships between stress, arousal, and disinhibition.

The effects of arousal on disinhibition suggest an alternative explanation for the increased disinhibition that is seen following exposure to substance-related cues, given that it is well-established that substance-related cues lead to increased physiological arousal (62). Therefore, the mechanism through which substance-related cues lead to increased disinhibition may be as simple as increased arousal, a hypothesis which should be investigated in future research. We conclude this section by noting that substance-related cues, stressors, and highly arousing emotional events (which could be either positive or negative) are known to precipitate relapse to drug-seeking (79) and state fluctuations in disinhibition are a plausible mechanism to explain these effects. However, to our knowledge no research has assessed whether fluctuations in disinhibition caused by substance-related cues, stress, or arousal directly mediate drug-seeking.

## Motivational Biases and Mental Sets

We have shown how environmental and psychological factors (ingestion of alcohol, ego depletion, substance-related cues, and stressors) can increase disinhibition. In this section we discuss “internal” factors, such as motivation and mental set, which are also known to alter disinhibition. Behavioral measures of disinhibition, in particular the Stop Signal task, set up a response conflict (a competition) between speed and accuracy (80). This response conflict can be experimentally manipulated, resulting in fluctuating disinhibition (81). For example, Leotti and Wager (82) propose that there are marked individual differences in the aversion to making mistakes, and these influence how a person would respond when completing a Stop Signal task. Imagine a person who is extremely averse to making mistakes: for this person, the conflict between responding rapidly and avoiding inhibition errors would be tipped in favor of avoiding inhibition errors, and this person would have a strategic bias that means they would make (relatively) few inhibition errors if they were to perform a Stop Signal task. It is possible to alter participants’ strategic biases when they perform these tasks, for example by providing instructions and/or providing financial incentives that reward either rapid responding or successful inhibition. As an interesting aside, we note that similar incentives can overcome the effects of ego depletion (83) and psychomotor impairment after alcohol consumption (84), suggesting some degree of intentional control over the expression of disinhibited behavior (85).

Guerrieri et al. (86) and Jones et al. (87, 88) were able to influence participants’ responding during a Stop Signal task by manipulating task instructions. One group of participants were instructed to prioritize rapid responding rather than successful inhibition, whereas these instructions were reversed for a different group of participants. These instructions had the anticipated effects on performance on the Stop Signal task: the former group were faster to respond on “Go” trials but they made more inhibition errors, compared to the latter group, who were slow to respond on “Go” trials but were much more successful at inhibiting their responding. Arguably, this manipulation of task instructions changes participants’ mental set while they complete the Stop Signal task. In the former group, a disinhibited mental set is temporarily induced, whereas a more restrained and cautious mental set is induced in the latter group. Once these disinhibited mental sets have been induced, this allows us to investigate whether disinhibited (versus restrained) mental sets have a causal influence on craving and food or alcohol consumption, something which was accomplished by measuring these things immediately after participants had completed the Stop Signal task. The findings were clear: participants in whom a disinhibited mental set had been induced subsequently consumed more food (86) or beer (87, 88) compared to participants in whom a restrained mental set had been induced. A follow-up study demonstrated that these differential task instructions also influenced an electrophysiological index of (dis)inhibition, the amplitude of the P300b event-related potential (66). In all of the alcohol studies (66, 87, 88), individual differences in disinhibition (both behavioral measures and their electrophysiological correlates) were positively correlated with consumption of beer: those participants who were most “disinhibited” after this manipulation of mental set, consumed the most beer immediately afterward. However, despite influencing drinking behavior, these task instructions had no effect on subjective alcohol craving in any of these studies. Overall, these studies provide direct support for the notion that a disinhibited mental set can lead to increased alcohol consumption, at least in laboratory settings. Importantly, this is not the same as directly “training” participants to act in a more or less disinhibited way, but this is an issue that we address in the following section.

## Boosting Self-Control: Can We Train People to be Less Disinhibited?

To summarize so far, we have demonstrated that disinhibition is an important feature of substance use disorders that may occur pre-morbidly to and serve as a risk-factor for the development of those disorders. Furthermore, various environmental and psychological variables are known to produce transient changes in disinhibition, and this fluctuating disinhibition may exert a causal influence on craving and substance consumption in the laboratory. From the clinical perspective, how can we exploit this knowledge in order to develop novel treatments for substance use disorders?

One novel approach to treatment would be to directly target the cognitive processes that have a causal influence on substance self-administration. This treatment strategy has the potential to complement (but not replace) existing approaches based on pharmacotherapy and “talking therapies” (89, 90). Despite this being a relatively underdeveloped area of research, some forms of this “cognitive training” appear to be effective in alcohol dependence (91, 92). This raises the question of whether inhibitory control can be directly trained. One approach is to require patients to repeatedly practice self-control tasks, something which should (in theory) increase the strength of the inhibitory control (or self-control) “muscle,” according to the resource model of self-control. Some studies have demonstrated that improving general control processes can lower alcohol consumption and reduce the risk of smoking relapse (93, 94). In more general terms, Hagger et al. (48) reported an overall large effect size for the effects of self-control training on health-related behaviors.

Working memory is important for maintaining task goals and updating information, which are considered important components that underlie self-control and effective inhibition (95, 96). Indeed, there is a large amount shared variance between the working memory and inhibitory control. Training working memory has been shown to reduce alcohol consumption at 1-month follow-up (97) and a combination of working memory and inhibition training improving health-related outcomes in obese children (98). Furthermore, training working memory have been shown to transfer to improvements in other cognitive tasks (99). It is therefore possible that the effects of working memory training on health-related outcomes may by partially attributable to improvements in inhibitory control. However, this issue has yet to be investigated.

In addition to training general control mechanisms, some recent studies have demonstrated that it may be possible (and beneficial) to train participants to improve their response inhibition specifically when they are faced with substance-related cues. In two studies, Houben and colleagues (100, 101) required their participants to perform a Go/No-Go task in which alcohol-related and neutral cues were embedded. One group of participants were always required to inhibit (“No-Go”) when alcohol-related pictures were presented, and they always had to respond (“Go”) when neutral cues were presented. In a different group, these contingencies were reversed. The primary finding was that, at 1-week follow-up, participants who had consistently inhibited their responses when presented with alcohol cues reported significantly reduced alcohol consumption, compared to the group who had consistently inhibited their responses when neutral cues were presented. However, there were no significant effects on alcohol self-administration in the laboratory when measured immediately after the end of training. These results were replicated in the second study (no immediate effects on alcohol consumption, but a clear reduction in self-reported alcohol consumption at follow-up after 1 week). In the second study, the authors also examined what mediated the effects of the training on alcohol consumption, and they found that the reduction in drinking was mediated by changes in automatic affective associations for alcohol cues rather than the (predicted) improvements in response inhibition. These findings support theories of *inhibitory devaluation*, which suggests that a conflict between wanting to respond to positive stimuli but having to inhibit is resolved by a devaluation of these stimuli (102, 103). This phenomenon has been applied to other hedonic stimuli such as food (104) and sexual images (105).

Jones and Field (106) used a similar training procedure, this time embedded in a Stop Signal task, in an attempt to extend the findings from the studies reported by Houben et al. (100, 101) and to try to identify an inhibition training procedure that may exert its beneficial effects by bolstering inhibitory control (rather than by changing affective associations with alcohol). Participants completed a Stop Signal task in which alcohol-related and neutral pictures were shown; participants were required to categorize the pictures as quickly as possible but to inhibit their responding whenever they heard a tone. In one group of participants, the majority of stop signal tones occurred during presentation of alcohol pictures, whereas for another group of participants the tone was paired with neutral pictures instead. Results indicated that the group that inhibited mainly to alcohol cues showed a progressive decrease in inhibition errors and slowing of reaction times to alcohol cues, over the course of multiple blocks of the task. Importantly, immediately after the training this group consumed less beer than participants in the control group, which suggests that this inhibition training – in which participants learn to inhibit their responding but only when alcohol-related pictures are presented on the computer screen – can have beneficial effects on drinking behavior. However, unlike in the studies reported by Houben et al. (100, 101), these beneficial effects of training were not evident at the 1-week follow-up. The immediate effects of cue-specific inhibition training on *ad libitum* alcohol consumption in the laboratory were replicated by Bowley et al. (107), who used a modified Go/No-Go task similar to that used by Houben et al. (100, 101). Those authors found that the reduction in alcohol consumption was comparable to that seen in an active control group that received a brief (5 min) alcohol intervention. However, this study also failed to demonstrate beneficial effects of the training at 1-week follow-up.

At both Liverpool and Maastricht we are currently working on studies with more intensive training, conducted over multiple sessions and with longer follow-up periods, in an attempt to identify the optimum parameters for inhibition training to produce immediate reductions in alcohol consumption in the laboratory, together with reductions in drinking that can be maintained over the longer term. If further work suggests that this type of inhibition training is effective, it may work in a similar way to other types of cognitive training, by giving participants a few moments to resist their powerful tendencies to use the substance and to engage a more appropriate coping response instead [see (90)].

Beliefs about self-control: why we should be careful what we tell people about the role of disinhibition.

Whilst attempting to bolster inhibitory control may ultimately lead to a novel, effective method of treatment for substance use disorders, it is imperative that individuals are motivated to change their behavior. Many current treatments such as motivational interviewing and cognitive behavioral therapy aim to increase the motivation to change substance use behavior, and furnish patients with the skills needed to bring about and then maintain this behavior change (108, 109). As well as motivation to change behavior (i.e., exercise restraint), individuals’ beliefs about their ability to inhibit their behavior may be important not only in terms of their substance use but also in terms of the applicability of these proposed interventions. Whilst it is generally acknowledged that having some confidence in the ability to control substance use is likely to increase the chances of maintaining abstinence, the evidence that this plays a major role is rather weak (110) and indeed, excessive self-efficacy (“over-confidence”) may be detrimental to the chances of long-term success (111). Over-confident individuals may exhibit a “restraint bias,” whereby unrealistic beliefs about the ability to control substance-seeking behavior may lead people to expose themselves to increased temptation, which results in substance use (112, 113). Therefore, beliefs about the ability to exercise restraint (or overcome disinhibition) may have a direct influence on substance-seeking behavior, but these beliefs might also moderate the influence of both alcohol intoxication and ego depletion on disinhibited behavior, as discussed in previous sections of this paper.

## Conclusions and Future Directions

The purpose of this review was to identify the environmental and psychological triggers that may cause disinhibition to fluctuate, and outline the implications of these fluctuations for the understanding and treatment of substance use disorders. The focus on state disinhibition is timely, because theoretical models now acknowledge that disinhibition is unlikely to function as a fixed and stable trait. Instead, temporary fluctuations in disinhibition may also contribute to substance use (2). The research reviewed in this paper lends support to this idea.

**Figure 1:**
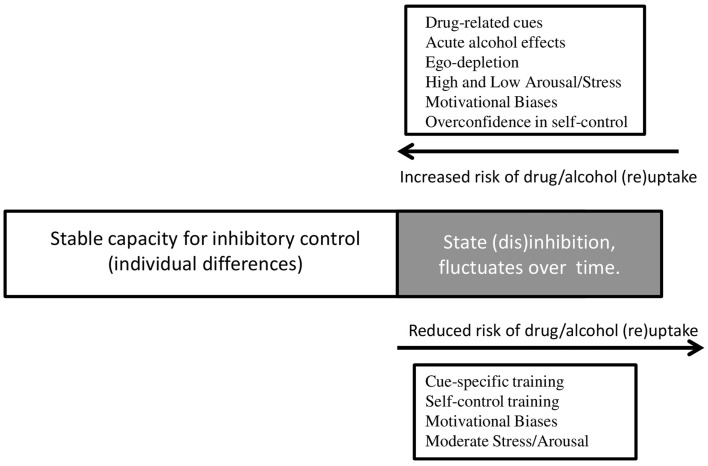
**Environmental triggers and psychological processes that underlie changes in state disinhibition, and their influence on substance use**. Individuals have a stable capacity for inhibitory control, but a component of this capacity appears to fluctuate in response to environmental triggers and psychological processes. If individuals are in a disinhibited state, they are more likely to engage in substance use, or relapse to substance use after a period of abstinence. If individuals experience a decrease in state disinhibition, they are less likely to engage in substance use, or relapse. Drug-related cues, alcohol intoxication, ego depletion, arousal, stress, motivational biases, and over-confidence all lead to increased disinhibition and thereby increase the risk of substance use. Disinhibition can be reduced after different types of cognitive training, by motivational biases, and by moderate levels of stress and arousal – resulting in a reduced risk of substance use.

Given the emerging evidence discussed in this review, we propose a theoretical framework (visualized in Figure 1) that extends the hypothesis formulated by de Wit (2). We argue that individuals have a relatively stable *capacity* for inhibitory control, on the basis of research suggesting a general stability of inhibitory control throughout adulthood following maturation of the prefrontal cortex during adolescence (114, 115). This capacity is likely to be determined by a host of heritable and environmental factors which we have not addressed in detail here [but see (7, 116)], and like all human characteristics there is likely to be wide individual variation. It is also likely that this capacity may be reduced by neurotoxic effects of chronic substance use, particularly if this occurs during adolescence. Such neurotoxic effects have not yet been convincingly demonstrated but they are biologically plausible, and difficult to rule out on the basis of the research that is currently available [see (117)].

Within this relatively stable *capacity* for inhibitory control, we see “state” fluctuations in response to environmental triggers and motivational factors, as outlined in this review. Fluctuations in this state may increase the risk of substance use at particular times and in particular contexts, but the magnitude of these fluctuations (and their influence on behavior) is constrained by individual differences in the capacity for inhibitory control. Overlaid on top of this, the individual’s *beliefs* about disinhibition and self-control can directly determine their behavior, including substance use. For example, individuals have beliefs about their ability to control behavior in the face of temptation [restraint beliefs c.f. (112, 113)], about the effects of alcohol intoxication on disinhibition [intentionality c.f. (85)], and about the effects of self-control depletion on future behavior (59). We hypothesize that beliefs may interact with the capacity for inhibitory control, but they may also influence substance use independently as demonstrated by Jones et al. (112), and Nordgren et al. (113).

Priorities for future research include the identification of other factors that influence state fluctuations in disinhibition, and characterization of individual differences that may moderate these effects, e.g., personality constructs such as “ego-control” and “ego-resiliency” (118, 119). Another important line of research is the development of new psychological interventions for substance use that can improve inhibitory control and mitigate the influence of environmental and motivational factors on state disinhibition. Whilst the recent studies have established proof-of-concept, it is unknown whether repeated inhibitory control training could promote long-lasting changes in drinking or other substance use. A recent review of the available literature suggests that inhibitory control may be “subject to fast plastic changes” (120). Therefore, it is possible that multiple sessions of inhibition training may increase an individual’s capacity for inhibitory control, allowing for more behavioral flexibility when faced with the situational, psychological, and environmental factors discussed in this review. Therefore, inhibition training may provide a non-invasive adjunct to existing treatments [c.f. the effects of repeated working memory training (101) and approach bias training (92)].

Finally, future research should focus on improving the efficacy of this training and identifying the mechanism(s) of effect. Firstly, a comparison of different training mechanisms is needed as both the stop signal and Go/No-Go tasks are thought to measure subtly different forms of inhibition (action cancelation and action restraint, respectively). Therefore, modified versions of these task used for training are thought to improve automatic inhibition and controlled inhibition respectively [see explanations by (106, 120)]. It might be that training both forms of inhibition provides the most protection against state fluctuations. Secondly, the generalizability of inhibition training is currently unclear: data from Jones and Field (106) and Bowley et al. (107) suggest the effects of training did not generalize to drinking outside of the laboratory, whereas data from Houben et al. (100, 101) were more promising. Therefore, future research should attempt inhibition training in situations where individuals are likely to drink or in as many different contexts as possible, and it could be administered on a tablet computer or smartphone in naturalistic environments.

However, we emphasize that clinicians should employ caution when attempting to strengthen inhibitory control. Over-confidence in the ability to control behavior can be damaging and lead individuals to expose themselves to greater levels of temptation and increase their substance use. It is important to identify the optimum balance between the capacity for self-control, motivation to change behavior, and beliefs about the ability to exercise self-control. Finally, we note that the majority of research on this topic that has been conducted to date has investigated alcohol use disorders, and there is a pressing need to investigate whether these principles apply to other substance use disorders as well.

To conclude, in this review we have identified the environmental triggers and psychological processes that can cause disinhibition to fluctuate, and shown that these fluctuations can increase substance use. Further research is needed to clarify the psychological mechanisms that underlie these effects, and to exploit this knowledge in order to develop new psychological treatments for substance use disorders.

## Conflict of Interest Statement

The authors declare that the research was conducted in the absence of any commercial or financial relationships that could be construed as a potential conflict of interest.
